# Measurement Invariance of the Burnout Assessment Tool (BAT) Across Seven Cross-National Representative Samples

**DOI:** 10.3390/ijerph17155604

**Published:** 2020-08-03

**Authors:** Leon T. de Beer, Wilmar B. Schaufeli, Hans De Witte, Jari J. Hakanen, Akihito Shimazu, Jürgen Glaser, Christian Seubert, Janine Bosak, Jorge Sinval, Maksim Rudnev

**Affiliations:** 1 WorkWell Research Unit, Potchefstroom Campus, North-West University, Potchefstroom 2531, South Africa; 2 Research Unit Occupational & Organizational Psychology and Professional Learning KU Leuven, 3000 Leuven, Belgium; w.schaufeli@uu.nl (W.B.S.); hans.dewitte@kuleuven.be (H.D.W.); 3 Department of Psychology, Utrecht University, 3584 CS Utrecht, The Netherlands; 4 Optentia Research Focus Area, Vanderbijlpark Campus, North-West University, Vanderbijlpark 1900, South Africa; 5 Workability and Work Careers, Finnish Institute of Occupational Health, 00032 Helsinki, Finland; Jari.Hakanen@ttl.fi; 6 Department of Policy Management, Keio University, Fujisawa 252-0882, Japan; ashimazu-tky@umin.ac.jp; 7 Department of Psychology, University of Innsbruck, 6020 Innsbruck, Austria; juergen.glaser@uibk.ac.at (J.G.); christian.seubert@uibk.ac.at (C.S.); 8 Business School, Dublin City University, Dublin 9, Ireland; janine.bosak@dcu.ie; 9 Business Research Unit (BRU-IUL), Instituto Universitário de Lisboa (ISCTE-IUL), 1649-026 Lisbon, Portugal; jorgesinval@gmail.com; 10 William James Center for Research, ISPA, Instituto Universitário, 1149-041 Lisbon, Portugal; 11 Faculdade de Medicina, Universidade de Lisboa, 1649-028 Lisbon, Portugal; 12 National Research University Higher School of Economics, Moscow 101000, Russia; mrudnev@hse.ru

**Keywords:** burnout, measurement invariance, work stress, work overload, work-related well-being, structural equation modeling

## Abstract

The aim of this study was to investigate the measurement invariance of the Burnout Assessment Tool (BAT) across seven cross-national representative samples. In this study, burnout was modeled as a second-order factor in line with the conceptual definition as a syndrome. The combined sample consisted of 10,138 participants from countries in Europe and Japan. The data were treated as ordered categorical in nature and a series of models were tested to find evidence for invariance. Specifically, theta parameterization was used in conjunction with the weighted least squares (mean- and variance adjusted) estimation method. The results showed supportive evidence that BAT-assessed burnout was invariant across the samples, so that cross-country comparison would be justifiable. Comparison of effect sizes of the latent means between countries showed that Japan had a significantly higher score on overall burnout and all the first-order factors compared to the European countries. The European countries all scored similarly on overall burnout with no significant difference but for some minor differences in first-order factors between some of the European countries. All in all, the analyses of the data provided evidence that the BAT is invariant across the countries for meaningful comparisons of burnout scores.

## 1. Introduction

Since the term’s initial appearance in the 1970s [1], burnout has become an increasingly popular occupational health concern, not only known to the academic and medical communities but also increasingly used by the public in general conversation. Research over the past decades has shown that burnout has a host of negative consequences for individuals (e.g., Type 2 diabetes, coronary heart disease and severe injuries), organizations (e.g., absenteeism, poor performance and job dissatisfaction) and society at large (e.g., early mortality, hospitalization and disability pensions; for a review, see: [2,3]). Burnout has also been connected to the quality of care provided by healthcare professionals (e.g., nursing) [4,5,6]. Moreover, research has firmly established that burnout and its consequences exist in various occupations and environments [7,8]. The growing importance of burnout is also reflected by its recent inclusion as an “occupational phenomenon” in the latest version of the International Classification of Diseases (ICD-11) of the World Health Organization [9].

From the outset in the early 1980s, the measurement of burnout has been dominated by the Maslach Burnout Inventory (MBI; [10]), which is used in about 90% of all empirical papers on the subject [8]. Hence, the MBI serves as the gold standard to assess burnout, which effectively means that burnout is what the MBI measures. However, this circularity and mutual dependence of concept and assessment may impede new and innovative research that would lead to a better understanding of burnout. All the more because the MBI has been criticized on conceptual, practical, and psychometrical grounds. First, the MBI was developed inductively as a research instrument by factor analyzing a pool of items that were formulated, based on in-depth interviews with professionals working in the human services [11]. Therefore, no conceptual basis exists for the three dimensions that are included in the MBI (i.e., exhaustion, depersonalization/cynicism and lack of professional efficacy) [12]. Indeed, some studies have shown that the core of MBI-measured burnout is more accurately captured by exhaustion and depersonalization/cynicism with (a lack of) professional (in)efficacy being a separate component [13]. Moreover, professional inefficacy has also been argued to be either a cause or a consequence of burnout (e.g., [12]). Burnout has also been found to be closely associated with a decline in cognitive functioning, specifically: executive functions, attention and memory [14]—indicating that the content of the MBI potentially needed updating. In sum, the validity of the three constituting elements of burnout that are tapped by the MBI is questioned.

Secondly, the practical use of the MBI is hampered by: (a) the lack of clinically validated cut-off values [15]; (b) the lack of statistical norms that are based on national representative samples [16] and (c) the fact that it yields three different subscale-scores instead of a single burnout score [17]. It is important to note that—as mentioned above—the MBI was developed as a research instrument rather than an assessment tool, which also limits its practical usability.

Thirdly, on the technical side the MBI has been criticized for: (a) skewed answering patterns that may affect its reliability [18]; (b) including positive (professional efficacy) items to assess a negative state [19,20] and (c) inconsistent factorial validity, that is, the three-factor structure is not always replicated. Although there is substantial support for the original three-factor structure of the MBI [21], also two [22], four [23] and five factors have been found [24]. Moreover, it appears that at least three items ( ‘I feel very energetic’, ‘Working with people directly puts too much stress on me’ and ‘I just want to do my job and not be bothered’) are unsound and should be removed [25,26]). Finally, a recent study found that the three-factor structure of the MBI was invariant across countries, but only after six of the 22 items had been deleted [27], thus calling the cross-national factorial validity of the original MBI into question. In a similar vein, a 12-country comparative study showed that seven MBI items are problematic from a cross-cultural point of view [28].

These conceptual, practical and psychometric criticisms call for an alternative self-report burnout instrument. This critical call for a new instrument has been recently addressed by the introduction of the burnout assessment tool [29,30]. The conceptual basis of the BAT builds on the analysis of Schaufeli and Taris (2005), who argued that occupational fatigue represents both the inability and the unwillingness to spend effort on work tasks, which is reflected by an energetic and motivational component, respectively [12]. The inability to spend effort manifests itself as exhaustion (e.g., feeling extremely tired, worn-out and depleted), whereas the unwillingness to perform manifests itself as mental distancing (e.g., increased resistance and aversion to work, lack of interest and disengagement). Thus, inability (exhaustion) and unwillingness (distancing) are the key components that constitute two sides of the same burnout coin [12]. Based on in-depth interviews with professionals who work on a daily basis with persons who suffer from burnout (such as general practitioners, occupational physicians, occupational health psychologists and career counselors), two additional core dimensions of burnout were uncovered: emotional impairment and cognitive impairment [29,30,31]. The former refers to the reduced functional capacity to adequately regulate one’s emotional processes such as anger or sadness, whereas the latter refers to the reduced functional capacity to adequately regulate one’s cognitive processes, such as memory or attention. The functional capacity to regulate one’s emotional and cognitive processes is reduced because all energy is drained; in that sense, exhaustion is paramount in burnout. In addition, reduced professional efficacy was not identified as a constituting element of burnout in the in-depth interviews of the professionals. Therefore, in this new conceptualization and based on past research the professional (in)efficacy component has been excluded as a core component of the burnout syndrome [29,30].

Meanwhile, this novel burnout instrument, labeled the burnout assessment tool (BAT), was studied extensively and showed good validity evidence in two countries: Belgium (i.e., the Dutch speaking Flemish region) and The Netherlands [30]. More particularly, a second-order model with all four first-order factors (exhaustion, mental distance and cognitive and emotional impairment) explained by one general, second-order, burnout factor was successfully fitted to the data. All four subscales also showed good internal consistency and stability across time. Using a multitrait-multimethod analysis, convergent validity was shown with two other burnout measures (MBI; [32]) and Oldenburg Burnout Inventory (OLBI; [33]) and divergent validity with measures of workaholism and boredom, respectively. Finally, because the BAT was associated with various job demands, job resources and personal resources in ways as predicted by the job demands–resources model [34], content validity of the BAT was shown as well.

In this paper we focus on the measurement invariance of the BAT from a cross-national perspective. Like the MBI, the BAT is a multi-dimensional burnout instrument, but in contrast to the MBI the BAT assumes that burnout is a *syndrome*, meaning that all four dimensions are interrelated and refer to the same underlying condition; i.e., burnout. Tellingly, the MBI test-manual explicitly states: “In general, each respondent’s scale scores should be calculated and interpreted separately. Note that responses to MBI items should not be combined to form a single ‘burnout’ score” [32] (p. 44). The fact that the BAT conceptualizes burnout as a syndrome means that a second-order factor model is warranted in order to model this conceptualization, and as outlined above, is expected to fit the data not only in Dutch speaking countries but also across nations because the BAT is developed for the use in different national and cultural settings [29]. Although some studies have been carried out on the cross-cultural factorial invariance of the MBI (e.g., [26,27]), to the best of our current knowledge, none of these used nationally representative samples. As Aboagye et al. have pointed out [27], this might be the reason for the relatively poor cross-national validity evidence of the MBI; namely, its factorial invariance depends for a large part on the sociodemographic and occupational context. Hence, by comparing non-representative national samples, some occupational settings or sociodemographics will be over- or under-represented. For that reason, we will use national representative samples from seven different countries: The Netherlands, Belgium (i.e., Flanders), Germany, Austria, Ireland, Finland and Japan.

Another major consequence of not using representative samples in previous studies with the MBI is the implication that levels of burnout cannot be accurately compared across nations. It is striking that even forty years after the introduction of the MBI not a single systematic comparison of burnout levels between countries has been carried out using nationally representative samples. This means that we still do not know whether burnout levels in one country are higher than in another. To the best of our knowledge, the current study is the first to allow a systematic comparison of burnout levels among multiple countries.

In sum, our main research questions are: (a) Is BAT-assessed burnout, conceptualized as a second-order factor, invariant across seven different countries? (b) What are the cross-national levels of burnout, under the assumption that (a) is answered affirmatively.

## 2. Materials and Methods

### 2.1. Participants

The combined total sample (*N* = 10,138) consisted of data from seven nationally representative samples provided by the BAT research consortium, which includes a network of burnout researchers working on the BAT from across the world. Below a breakdown is provided per country and where percentages do not add to a 100% it is due to rounding and/or missing values.

Specifically, the Dutch (*n* = 1500; M_age_ = 41.26, SD = 13.36; male = 54.10%, female = 45.90%) and the Belgian (Flanders; *n* = 1500; M_age_ = 41.37, SD = 11.46; male = 54.30%, female = 45.70%) samples were randomly drawn from the Flemish and Dutch labor force by a commercial surveying agency (iVox) in such way that they were representative of age, gender and industry. The selection criteria for age and gender were ‘hard’ criteria, meaning that they must perfectly match the distribution of the workforce, whereas for industry a ‘soft’ selection criterion was used, which allowed a maximum deviation of 10% from the population. In terms of the sector breakdowns, most participants in both samples were sampled from the services sector (Dutch = 47.00%; Belgian = 33.10%), followed by the industrial sector (18.50%; 19.90%). The remaining sectors consisted of the primary sector (3.60%; 0.70%), government and public administration (9.50%; 18.90%), health care and social services (14.30%; 14.70%) and education (7.20%; 12.70%).

For Austria (*n* = 1059; M_age_ = 42.98, SD = 13.32; male = 50.10%, female = 49.90%) and Germany (*n* = 1073; M_age_ = 41.79, SD = 13.14; male = 51.50%, female = 48.50%), random samples were drawn from the Austrian and German labor force by a commercial surveying agency (Respondi). Both samples were representative of gender by age groups, with an observed maximum deviation of 3.7% (0.5%) from the population in Austria and Germany. Participants represented a broad range of economic sectors, i.e., health care, social services and law enforcement (Austria: 13.50%; Germany: 13.40%); retail, wholesale and repair (11.50%; 9.50%); commercial services (10.60%; 10.50%); education (9.30%; 6.80%); public administration and governance (8.80%; 11.00%); manufacturing (8.10%; 10.30%); banking, real estate and financial services (5.20%; 5.10%); hospitality (4.6%; 2.1%); transportation, storage and distribution (3.6%; 4.8%); arts, entertainment, recreation and sports (3.6%; 3.5%); construction (3.3%; 5.6%); agriculture, forestry and fishery (1.60%; 0.50%) and other sectors (16.2%; 17.0%).

For Finland (*n* = 2299; M_age_ = 43.50, SD = 11.34; male = 49.70%, female = 50.30%) the sampling was done via an online survey that was distributed to employees in 34 Finnish municipalities as part of a longitudinal project, the current BAT data for Finland was collected in the second wave of measurement as the scale was not yet available in the first wave. Roughly half of the sample worked in education and social and health services, as 15.00% of the participants worked in education (i.e., pre-, primary and secondary school), social services (13.00%; e.g., child protection services, elderly care, services for the disabled, etc.), municipal health care services (12.00%; e.g., medical centers, mental health care services, hospitals, home care and dental care), municipal daycare services (10%), school services (5.00%), general administration (9.00%), technical services (8.00%; e.g., building inspection, zoning of land, public transport, environment, recycling, waste management, etc.) and culture and recreation services (8.00%; e.g., libraries, sports services, etc.). The remaining (20.00%) reported working in other support services across these sectors.

For Ireland (*n* = 431; M_age_ = 42.10, SD = 12.30; male = 53.60%, female = 46.40%) was drawn from a larger heterogeneous sample (*N* = 1101), which was achieved by asking undergraduate students to reach out to and invite four working individuals they know to participate in the study, complemented with participant responses from the Irish coordinator’s own network and industry contacts. For the Irish sample a representative sample was drawn from a larger sample in terms of age and gender. Participants in the Irish sample came from a broad range of sectors, i.e., education (13.00%), banking, real estate and financial services (12.50%), health care, social services and law enforcement (10.90%), commercial services (10.70%), retail, wholesale and repair (9.00%), public administration and governance (7.20%), construction (5.10%), manufacturing (4.40%), hospitality (4.20%), agriculture, forestry and fishery (2.80%), arts, entertainment, recreation and sports (2.10%), transportation, storage and distribution (2.10%) and other (15.50%). Two participants did not answer the question (0.50%), and similarly for the Finnish sample weighting was assigned for the analyses to ensure that the sample was representative by age and gender.

For Japan (*n* = 1032; M_age_ = 40.24, SD = 11.69; male = 50.00%, female = 50.00%) samples were randomly drawn from the Japanese labor force by a commercial surveying agency (Macromill). Participants were equally allocated by gender and generation (ages 20–29, 30–39, 40–49 and ≥50). Respondents who met the inclusion criterion (full-time employment under 64 years old) were used in the analyses. Regarding marital status, almost half of the participants in the Japan sample were married (50.90%) followed by never married (39.40%). Mean hours worked per week was 40.10 (SD = 18.5). Regarding occupation, 36.10% worked for clerical work, 21.30% for professional and engineering, 12.20% for sales and 10.20% for manufacturing.

### 2.2. Measure

The BAT was used to measure burnout conceptualized as a syndrome comprising four components (exhaustion, emotional impairment, cognitive impairment and mental distance) [29,30]. Specifically, exhaustion (EX) was measured by eight items (e.g., ‘When I get up in the morning, I lack the energy to start a new day at work’), emotional impairment (EI) with five items (e.g., ‘At work I may overreact unintentionally’), cognitive impairment (CI; e.g., ‘At work I struggle to think clearly’) with five items and mental distance (MD) was also measured by five items (e.g., ‘I feel indifferent about my job’). Table 1 below presents the *ordinal* Cronbach’s alpha [35] values for each of the components, as well as for the total BAT score in each of the countries.

As can be seen all the values were above 0.70 and exceeded 0.80 providing evidence of excellent internal consistency of the scales.

### 2.3. Data Analysis

**Figure 1:**
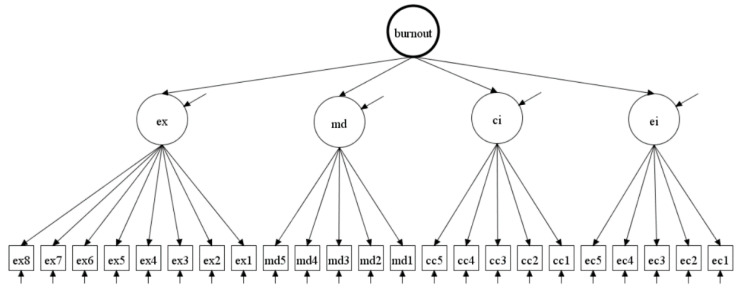
The second-order model of burnout based on the conceptualization of the BAT. Note: ex = exhaustion, md = mental distance, ci = cognitive impairment, ei = emotional impairment.

Various descriptive statistics were calculated and included as an addendum to the article for those interested (see Supplementary Materials). As the concept of burnout, as assessed with the BAT is hierarchical, it consists of one overall burnout factor and four specific aspects of burnout. We therefore specified a second-order factor model to represent it (see Figure 1). This second-order model is compatible with the theory of burnout as a syndrome. To test the measurement invariance of the BAT, Mplus 8.4 was used to analyze the data [36]. First, the second-order model was tested in each country individually. Second, a series of multiple group CFA models were fitted in order to test for measurement invariance of the BAT. The items were treated as ordered categorical in nature as the assumption that the distances between response options are the same was not considered accurate, and therefore thresholds were estimated instead of intercepts. Specifically, the logit ordered categorical model with Theta parameterization [37] was used in conjunction with the weighted least squares estimator (mean and variance adjusted (WLSMV); see [38]).

In order to make an inference about invariance, usually a series of models with an increasing number of constraints is used and then these models are compared to each other. Models with categorical indicators can distinguish a configural invariance model, which tests an overall pattern of factor loadings; threshold invariance, which tests for equivalence of indicator thresholds, and scalar, or full invariance, which tests for both thresholds and factor loadings equivalence across groups [37,39]. Configural invariance allows one to compare signs of correlation and regression coefficients across groups. Scalar invariance allows comparing latent means across groups. However, as there is no agreement in the literature of what should be tested first (i.e., loadings or thresholds), we omitted separate tests of invariance and tested both in a single step. The second-factor model adds to these levels another two: a metric and scalar invariance of the second-order factor. Metric invariance allows comparison of correlation and regression coefficients involving the second-order construct; and scalar invariance allows comparison of latent means of the second-order construct. Since there are no guidelines for testing measurement invariance of the second-order factor models with categorical outcomes, we followed modified cut-off values for comparing models with categorical data: a change in comparative fit index (CFI) of 0.008 and root mean square error of approximation (RMSEA) of 0.060 for comparison of configural and scalar invariance of the first-order factors models (sum of the cut-off values suggested for the testing metric and scalar invariance) [39]. For testing the differences between models constraining the second-order part of the model, conventional criteria were used: a change in CFI and RMSEA of 0.015 [40]. If the models adhere to the guidelines (i.e., were smaller than these cutoff values), it indicates that the measure can be used in comparable ways between countries, as no substantial differences exist—indicating strong measurement invariance (see Supplementary Materials for output files based on the analyses).

There were several difficulties in the identification of the models, because in order to test invariance of the model with categorical indicators, which is the first-order part of our model, it is usually required to fix the latent means to zero [37]. However, in our case these first-order factors’ latent means were at the same time intercepts for the second-order factor, which we needed to test for equality [41]. In order to circumvent this problem and freely estimate the first-order latent means, the model was identified through fixing both first and second thresholds of one indicator per factor to be equal across groups. The covariance part of the model was identified using a marker indicator approach [42], that is, by fixing one loading per factor to 1.

For comparison purposes, the same country, Belgium, was used in all instances with no specific reason other than this was the area from where the BAT project originated.

## 3. Results

### 3.1. Testing the Second-Order Model in the Individual Countries

Table 2 below displays the fit statistics for each individual country based on the proposed second-order structure of the BAT (see Figure 1). This analysis was performed to provide an indication of the fit of the second-order model in each country before testing invariance. As a matter of fact, the fit of the model with the data in each country is a necessary (but not sufficient) requirement for invariance.

As can be seen, the second-order model had a good fit to the data in all countries, although the RMSEA for Germany, Ireland and Japan were above the proposed cut-off of 0.080. However, recent research has shown that the RMSEA can provide biased estimates in ordinal estimation and the SRMR should be considered more favorably [43]. The standardized root mean residual (SRMR) showed acceptable values (SRMR < 0.050) for these three countries. The remaining fit-statistic indicators all showed acceptable values (CFI ≥ 0.948; TLI [Tucker-Lewis index] ≥ 0.942). The analyses continued based on these results.

### 3.2. Measurement Invariance Testing Across Countries

Testing measurement invariance (MI) with ordered categorical indicators consisted of five phases: we started to evaluate the fit of a configural invariance model, which assumed that the overall factor structure is identical across countries but did not constrain any measurement parameters (model 1). Model 2 tested the invariance of the first-order factor loadings and thresholds were established. Model 3 tested invariance of the second-order factor loadings and models 4 and 5 tested equality of both loadings and intercepts of second-order factor. Table 3 presents the results of the analyses. 

In order to test scalar invariance of the second-order factors, and given that we were able to identify it only by fixing intercepts in one group to zero, the resulting model (model 4) did not truly assess scalar invariance, because the intercepts were fixed to be equal in all but one group. For this reason, we tested one extra model where all intercepts of the second-order factor were fixed to zero (model 5). This model is more restrictive than just equality of intercepts, however, this model also fit the data well.

Overall, the fit of all the models was high, CFI ranges from 0.979 to 0.975, RMSEA was between 0.055 and 0.064. Models with different across-group constraints showed a very similar fit to the data and the changes in fit indices were smaller than suggested cutoff values: CFI decreased at most by 0.003 and RMSEA increased at most 0.006. Therefore, we could conclude that both first- and second-order factors demonstrated scalar invariance.

### 3.3. Levels of Burnout Across Countries

Given the evidence for the invariance of the BAT, a final second-order multigroup model was specified to ascertain the overall fit of the second-order model with country as the grouping variable and without invariance constraints. The results showed that the model fitted well to the data (χ^2^ = 10,928.56, df = 2093, *p* < 0.001; CFI = 0.980; TLI = 0.983; RMSEA = 0.057; SRMR = 0.048). All the items loaded statistically significantly (*p* < 0.001) on the respective factors. Specifically, all groups showed item-loadings that were above 0.70 in the majority of instances. In all groups, as expected, the lower-order factors correlated positively with large effect sizes (*r*’s > 0.50).

Table 4 below provides the unstandardized latent means for the second-order burnout factor as well as for the first-order components in all countries as estimated by two different models (one for the second-order and another for the first-order factors). Standardized means are not presented because variances differ across countries, so the units in which these standardized means are measured can be ambiguous. This consideration is especially important regarding the first-order means, which are standardized both by their own variance and the variance of the higher-order factor.

**Figure 2:**
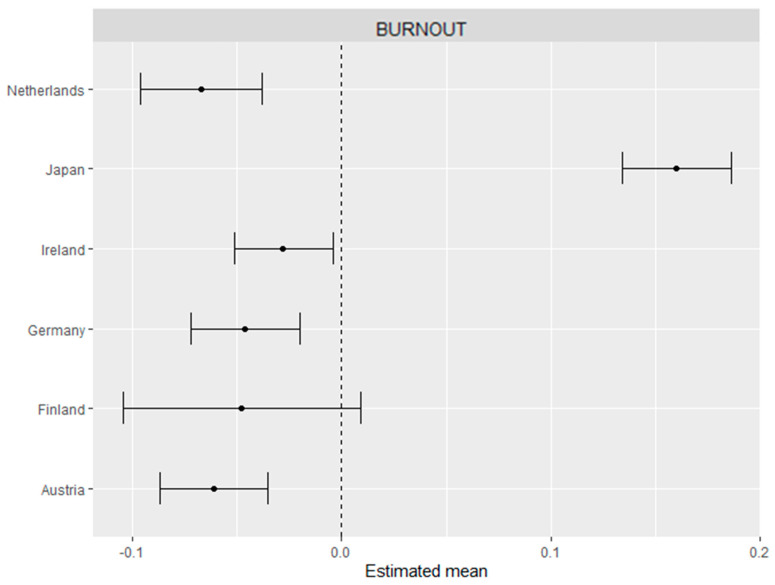
Effect sizes with 95% CIs for the second-order burnout factor with Belgium as a reference group.

**Figure 3:**
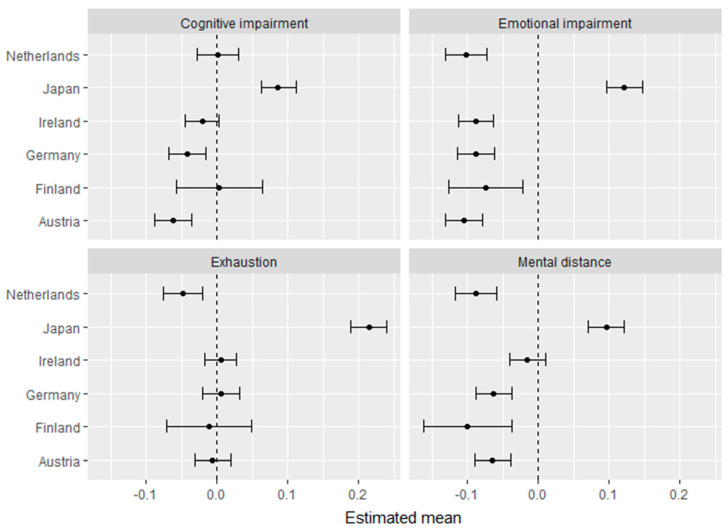
Effect sizes with 95% CIs for the first-order burnout component with Belgium as a reference group.

The scalar invariance established above allows comparison of latent means, however the absolute means say little about the degree of cross-country differences. In order to address this, and consider the differences, we computed effect sizes for each country. Specifically, two structural equation models were built with the countries as independent variables: the first model included the burnout factor as the dependent variable, and the second model used the first-order factors as dependent variables. Figure 2 and Figure 3 show the standardized regression coefficients plotted with 95% confidence intervals, which can be interpreted as effect sizes: Belgium (Flanders) is the reference group again as displayed by the dashed 0.0 line (see Figure 2 and Figure 3 below).

As can be seen from Figure 2 and Figure 3, all effect-size differences were very small, and the confidence intervals overlapped each other in most of the countries, with the largest deviation being Japan. The difference between Japan and the other countries is clearly shown for the second-order factor (i.e., the higher-order burnout score; Figure 2) and exhaustion (Figure 3). However, all European countries scored similarly on the second-order burnout factor (Figure 2), as all the 95% confidence intervals overlapped. In addition, slight differences between other European countries could also be observed, such as the Netherlands scoring somewhat lower than Ireland and Belgium on mental distance and Austria scoring lower than the Netherlands on cognitive impairment, respectively (Figure 3). Dutch workers also score slightly lower on exhaustion as compared to Irish workers.

## 4. Discussion

The aim of this study was to investigate the measurement invariance of the BAT, a new instrument to measure burnout conceptualized as a syndrome, across seven nationally representative samples. In addition, internal consistencies of the BAT and levels of burnout across these samples were compared. The results of the CFA analysis showed that the expected second-order burnout latent factor model based on the four latent components of the BAT (exhaustion, mental distance and emotional and cognitive impairment) was found to fit the data in the samples of all seven countries that were included in the current study. A subsequent series of model tests for measurement invariance using multi-group analyses showed that the four components were invariant across all countries. That is, strong measurement invariance: configural (similar factor pattern matrix), metric (similar factor loadings) and scalar (similar thresholds/intercepts) invariance existed across all countries. Moreover, the entire BAT as well as its four subscales were found to be internally consistent in all seven national samples. This indicates that: (1) the BAT is compatible with the notion that burnout can be modeled as a syndrome that consists of four interrelated symptoms that refer to a single underlying condition; (2) the BAT is a reliable burnout measure and (3) the BAT can be used in a comparable manner to measure burnout across nations.

The current study is unique in comparing burnout levels using nationally representative samples and showed that levels of burnout in Japan exceeded those of all six (European) countries. This might not come as a surprise given the pervasive culture of overwork in Japan, as is illustrated by the prevalence of “karoshi” (i.e., death from overwork) [44]. Indeed, it has been shown that Japan scores higher than the European countries on masculinity, which indicates a higher drive for competition, achievement and success [45]. A recent review also concluded that multiple, simultaneous actions need to be implemented by stakeholders in order to reduce overwork-related mental disorders, cerebrovascular and cardiovascular diseases in Japan [46]. One of the recent actions that have been implemented in April 2019 is legislation that regulates the maximum overtime of workers to 45 h a month and 360 h a year (with exceptions allowed up to 720 h a year) that came into effect [47]. Another sociocultural factor that could add to the speculation of why the difference in scores exists is individualism versus collectivism. Even though Japan scores higher than most other Asian counterparts on individualism, the score is lower than the European countries compared to in this study and a meta-analysis has shown that individualism is generally associated with more well-being [45,48]. However, these explanations remain somewhat speculative and further investigation is required.

Furthermore, our study revealed that, overall, burnout levels of the six European countries do not differ. Nevertheless, some minor differences between European countries exist in the first-order factors, whereby, specifically The Netherlands scored slightly lower than Ireland on mental distance and slightly lower than Ireland and a borderline case with Germany on exhaustion. Although the differences are very small, their pattern agrees with a recent study that showed that levels of work engagement were highest in the Netherlands compared with other European countries [16]. Since work engagement may be considered the positive antipode of burnout [49] it can be inferred that in countries with lower levels of burnout, levels of work engagement should be higher, and vice versa. Indeed, Japan has shown the lowest work engagement score in a study among 16 countries [50].

### Limitations and Recommendations

The current study is not without limitations. Although cross-sectional data is sufficient for measurement invariance, it would have been ideal to also consider the longitudinal invariance of the BAT in a test–retest type of analysis to gauge its stability. Furthermore, the current approach only considered seven countries with representative samples, but an extension of the analysis, particularly with countries from the America’s, Asia, Australia, and Africa should be considered to expand on its broader cross-national applicability, taking also more diverse cross-cultural setting into account. Therefore, it should also be mentioned that there were differences in the random sampling in some of the countries. So far it has proven to be challenging to find representative national samples, but that should not discourage investigating the comparability of the BAT with other (convenience) samples. Furthermore, the clear difference in scores between the European countries and Japan should be investigated in future research.

Other analytical techniques could also be considered to further delve into the psychometric properties of the BAT, such as approximate measurement invariance with a Bayesian approach, which allows for the specification of small-variance priors on the parameters. This approach is less restrictive and produces stronger invariance than the exact approach does [51]. Bayesian statistics is becoming a popular approach; however, it might be difficult to specify prior information for the parameters in an analysis of the BAT as it is a novel tool and not much published prior information is available for inclusion in the analyses. However, this should not discourage the possibility of using uninformed priors that might still serve the necessary function of a more flexible and practical analysis when sample size is sufficient [52].

## 5. Conclusions

The current study provides the strongest case so far for the cross-national invariance of a burnout measure. More specifically, this study used representative samples from the various countries that demonstrated that the BAT could be used to assess burnout in a similar way across various countries—considering burnout’s conceptualization as a syndrome theoretically and statistically. Moreover, it appeared that burnout is more prevalent in Japan compared to the European countries. Such international burnout comparisons are of growing importance in a globalizing world, and the BAT seems to be a promising research instrument to use for that purpose.

Individuals who want to plot their personal burnout risk scores against the data from the seven countries of the current article can do so at: https://theburnout.app.

## Figures and Tables

**Table 1:** Ordinal Cronbach’s reliability coefficients for the burnout assessment tool (BAT).

Country	EX	MD	CI	EI	BURNOUT
The Netherlands	0.91	0.93	0.93	0.95	0.96
Belgium (Flanders)	0.90	0.92	0.92	0.93	0.95
Germany	0.86	0.87	0.86	0.90	0.94
Austria	0.85	0.90	0.87	0.89	0.94
Ireland	0.84	0.87	0.88	0.86	0.92
Finland	0.84	0.90	0.88	0.81	0.91
Japan	0.91	0.80	0.91	0.91	0.95

Notes: EX = exhaustion, MD = mental distance, CI = cognitive impairment, EI = emotional impairment.

**Table 2:** Fit statistics for the second-order model in each country.

Country	df	χ^2^	CFI	TLI	RMSEA	SRMR
The Netherlands	226	1760.08	0.988	0.987	0.066	0.021
Belgium	226	2426.82	0.981	0.978	0.077	0.033
Germany	226	1817.08	0.961	0.957	0.081	0.037
Austria	226	1480.67	0.971	0.968	0.072	0.035
Finland	226	424.50	0.980	0.977	0.020	0.055
Ireland	226	866.76	0.964	0.960	0.081	0.049
Japan	226	3073.04	0.948	0.942	0.110	0.047

**Table 3:** Fit statistics for the models tested to investigate invariance across countries.

Model	χ^2^	df	CFI	RMSEA	ΔCFI	ΔRMSEA
1. Configural MI	10,444.46	1702	0.977	0.064	−	−
2. Full scalar MI of first order, configural MI of second order	11,179.02	2092	0.976	0.058	−0.001	−0.006
3. Metric MI of second-order factor, given scalar MI of the first-order factors	11,182.74	2110	0.976	0.058	0.000	0.000
4. Scalar MI of second-order factor, given scalar MI of the first-order factors	10,117.45	2119	0.979	0.054	0.003	−0.004
5. Second-order intercepts are fixed to zero (true second-order scalar model)	10,282.31	2122	0.978	0.055	−0.001	0.001

**Table 4:** Means (standard errors) of the latent variables as estimated in the analyses.

	AUS	BE *	FIN	GER	IRE	JAP	NL
*First-order model*							
Exhaustion	−0.03 (0.08)	0.00 (0.00)	−0.03 (0.10)	0.05 (0.07)	−0.07 (0.10)	1.12 (0.08)	−0.20 (0.07)
Emotional impairment	−0.72 (0.12)	0.00 (0.00)	−0.07 (0.11)	−0.60 (0.11)	−0.69 (0.14)	0.87 (0.11)	−0.39 (0.10)
Cognitive impairment	−0.30 (0.09)	0.00 (0.00)	0.11 (0.10)	−0.18 (0.09)	−0.68 (0.15)	0.49 (0.10)	0.11 (0.08)
Mental distance	−0.54 (0.11)	0.00 (0.00)	−0.34 (0.12)	−0.41 (0.10)	0.24 (0.18)	0.62 (0.09)	−0.45 (0.09)
*Second-order model*							
Burnout	−0.26 (0.07)	0.00 (0.00)	−0.09 (0.08)	−0.17 (0.07)	−0.15 (0.08)	0.71 (0.07)	−0.21 (0.06)

Note: * = Reference country Belgium (Flanders); AUS = Austria; FIN = Finland; GER = Germany; IRE = Ireland; JAP = Japan; NL = The Netherlands.

## Supplementary Materials

The descriptive statistics and Mplus output files are available online at https://osf.io/9cx4h/?view_only=868629cae91d4b3a9f1a7eed58553275.
